# Preference and performance of the green peach aphid, *Myzus persicae* on three Brassicaceae vegetable plants and its association with amino acids and glucosinolates

**DOI:** 10.1371/journal.pone.0269736

**Published:** 2022-12-01

**Authors:** Muhammad Afaq Ahmed, Ning Ban, Sarfaraz Hussain, Raufa Batool, Yong-Jun Zhang, Tong-Xian Liu, He-He Cao

**Affiliations:** 1 Key Laboratory of Insect Ecology and Molecular Biology, College of Plant Health and Medicine, Qingdao Agricultural University, Qingdao, Shandong, China; 2 State Key Laboratory for Biology of Plant Diseases and Insect Pests, Institute of Plant Protection, Chinese Academy of Agricultural Sciences, Beijing, China; 3 Key Laboratory of Agro-products Quality and Safety Control, Institute of Food Science and Technology, Chinese Academy of Agricultural Sciences, Beijing, China; Bangabandhu Sheikh Mujibur Rahman Agricultural University, BANGLADESH

## Abstract

The green peach aphid, *Myzus persicae* (Sulzer) is a generalist pest of various host plants, whose feeding preference and growth performance mainly depends on the quantity and quality of nutrients and defensive metabolites in host plants. Here, we studied the preference and performance of *M*. *persicae* on three major Brassicaceae vegetables in China and measured nutrient (amino acids) and defensive metabolites (glucosinolates) in these plants. We found that *M*. *persicae* preferred and performed better on Chinese cabbage than cabbage and radish, which may be due to the relatively higher concentration of amino acids and lower levels of indole glucosinolates in their leaves. The glucosinolates level in cabbage leaves was ten times higher than the other two plants, while the amino acid concentration in radish was only half of the cabbage or Chinese cabbage. The higher concentration of indole glucosinolates in cabbage and lower levels of amino acids in radish may account for the poorer preference and growth of *M*. *persicae* on these two plants. These results suggest that both amino acids and glucosinolates in plants may play important roles in the preference and performance of *M*. *persicae*, which provide new knowledge for the cultivation and breeding of Brassicaceae vegetables.

## Introduction

Aphids feed mostly on the phloem sap of their host plant, which is high in sucrose and lacking in essential amino acids, resulting in an unbalanced diet [1,2]. Aphids get most of their nitrogen and nutrients from free amino acids in phloem sap, and the concentration and composition of amino acids in phloem are essential indicators of nutritional quality for aphids [1,3]. Previous studies on the pea aphid (*Acyrthosiphon pisum*) and the bird cherry-oat aphid (*Rhopalosiphum padi*) showed that aphids fed on a chemically defined artificial diet with reduced amino acid concentration had lower survival, growth, and fecundity [4,5].

The green peach aphid, *M*yzus *persicae* (Sulzer) (Hemiptera: Aphididae), is an important economical polyphagous pest that infests more than 400 plant species, including Solanaceae, Cruciferae, and Leguminosae families [6]. *M*. *persicae* causes significant losses of crop yield by direct feeding and transmitting plant viruses [7,8]. Amino acids also play a major role in determining the aphid feeding rate, though sucrose is the main feeding stimulant for most aphid species [2,8,9]. For example, sucrose solutions containing glutamine, methionine, or valine were more attractive to *M*. *persicae* than sucrose solution alone, and *M*. *persicae* also preferred to feed and performed better on young cabbage leaves with more amino acids than older leaves [10]. Thus, more amino acids in plants generally enhance aphid feeding preference and growth performance [2,8,10]. During feeding, aphids also consume many phytotoxins such as cardiac glycosides, alkaloids, benzoxazinoids, and glucosinolates, which have toxic characteristics of inhibiting growth (antibiosis) or act as a feeding deterrent (antixenosis) against aphids [11,12].

The main phytotoxins in Brassicaceae plants are glucosinolates and especially their breakdown products, which generally reduce aphid feeding and growth [13,14]. *Myzus persicae* preferred to feed on Arabidopsis plants with lower glucosinolate content and aphids reproduction was negatively correlated with both indole and aliphatic glucosinolate content in defense-related Arabidopsis mutants [7,13,15]. However, other studies found no correlation between the glucosinolate content and *M*. *persicae* fecundity. For example, *M*. *persicae* still preferred and performed better on young cabbage leaves that contained a higher level of glucosinolates and amino acids [10]. These mixed results may be due to the inconsistence of absolute concentration and identity of glucosinolates among studies, as well as the neglected effects of nutrition. Nevertheless, the unstable indole glucosinolates added to artificial diet showed strong anti-feeding effects on *M*. *persicae* [13,15]. On the other hand, *M*. *persicae* has evolved some but not full ability to avoid the negative effects of glucosinolates. Therefore, the nutrition quality, glucosinolate identity and absolute concentration contribute to the outcome of aphid-plant interaction in Brassicaceae plants.

In this study, we first investigated the preference and performance of *M*. *persicae* on three most widely grown Brassicaceae crops Cabbage, Chinese cabbage, and radish in China and then monitored the feeding behavior of *M*. *persicae*. Radish has storage roots that absorb nutrients from leaves, which may imply a poor nutrition quality for aphids. Then, we measured the nutrition amino acid, sugar, and defensive metabolites glucosinolate in the leaves of these three host plants. This study not only explores the importance of both plant nutrients and defensive metabolites in plant-aphid interactions but also provides new knowledge for the cultivation and breeding of Brassicaceae crops.

## Materials and methods

### Plants and insects

Cabbage (*Brassica oleracea* L. var. ‘Jing Feng 1’), Chinese cabbage (*Brassica rapa pekinensis* var. ‘Dayu’), and radish (*Raphanus sativus* L. var. ‘Weixian K-57’) seeds were sown in the damped garden soil mixture in a controlled plant growth room at 22 ± 2°C with 50% relative humidity and 16:8 h L/D cycle. Plants at three weeks of age with three true leaves were used in all experiments. *Myzus persicae* were originally obtained from the sweet pepper plants in the field of Qingdao Agricultural University (Qingdao, Shandong, China), and were reared on each host plant for more than three months under the same conditions before experiment.

### Feeding preference of *M*. *persicae*

Leaf discs (2 cm in diameter) from the three plants were cut and placed equally apart inside one Petri dish (9 cm in diameter) layered with moistened filter paper to maintain the leaf turgor. Fifteen apterous adult aphids were placed in the center of each dish. The number of aphids that had settled on each leaf disc and did not move in 2 min was counted 1, 3, 8, 12, and 24 h after release. Ten replicates were performed for this assay as each dish was a replicate. This assay was replicated three times with aphids from one host plant each time.

### Performance assay

Three newly developed apterous adult *M*. *persicae* from each host plant were confined on the second true leaf of the respective host plant by the nylon mesh bag [8]. Adult *M*. *persicae* were removed from the leaves after 24 h, leaving five or six newborn nymphs on each leaf. After an additional ten days, the number of live aphids (both nymphs and adults) was counted and adults on each leaf were weighed in group on the microbalance (MSA 3.6 P-000-DM, resolution 0.001 mg, Sartorius, Gottingen, Germany). Each plant was as one replicate and ten replicates were performed for each host plant.

### Feeding behavior of *M*. *persicae*

The electrical penetration graph (EPG) direct-current system (Giga-8, Wageningen, Netherlands) was used to record *M*. *persicae* feeding behavior. A gold wire (18 μm in diameter) was attached to the dorsum of each adult aphid using silver conductive glue [16]. The plant electrodes were positioned into the soil of a potted plant, and the insect probes were fixed allowing for contact between the aphid and leaf surface. Feeding activities of aphids from respective hosts were recorded for 8 h at 24°C in a Faraday cage to avoid electromagnetic interference. Aphids were placed on the backside of a second true leaf of the respective host plant. The Stylet+d program was used to record the signal, and the Stylet+a software to label and analyze the EPG waveforms [17]. EPG parameters were calculated automatically by the EPG data 4.3 Excel workbook [18]. Each adult aphid and plant were used only once and were considered as one replicate. Overall, we obtained 15 successful replicates for each host plant.

### Amino acid extraction and analysis

Free amino acids were extracted by grinding 100 mg fresh leaf tissue with a glass mortar and pestle in 0.1 M HCl solution and were analyzed by the LTQ-XL linear ion trap mass spectrometer (LC-MS/MS; Thermo Fisher Scientific, Waltham-MA, USA) [19]. The XTerra MS C18 Column (125 Å pore size, 5 μm, 4.6 × 150 mm; Waters, Milford, MA, USA) was used for liquid chromatographic separations [20]. The elution of amino acids from the sample solution was conducted in a three-step gradient with mobile phase A (5% acetonitrile and 0.1% formic acid) and mobile phase B (100% acetonitrile). The spray voltage was fixed at 4.5 kV, while the temperature of the ion transfer capillary was set at 320°C. The masses of precursors and productions of each amino acid can be found in our previous work [20]. The external standard amino acid mixture of known concentrations (AAS18, Sigma-Aldrich, St. Louis, MO, USA) was used for quantification, accompanied by cysteine, tryptophan, asparagine, and glutamine [20]. Eight replicates were performed for each host plant.

### Sugar extraction and analysis

The sugars (fructose, glucose, and sucrose) were extracted from plants by grinding 100 mg fresh leaves in 1 ml 50% ethanol and were measured by the LTQ-XL linear ion trap mass spectrometer [8]. External standard sugars mixture was used for quantification. Overall, eight replicates were carried out for each host plant.

### Glucosinolate extraction and analysis

Fresh leaf (100 mg) was placed in a 1.5 mL centrifuge tube and kept in the boiling water for 2–3 min to deactivate the myrosinase activity in leaves [21]. The leaves were then ground in MilliQ water (1 mL) with a glass mortar and pestle and the mixture was then centrifuged for 20 min at 12,000 *g*, 4°C. Glucosinolates in the supernatant were measured by the LTQ-XL linear ion trap mass spectrometer [10,21]. The relative concentration of glucosinolates was determined by the standard curve made by 2-propenyl glucosinolate (sinigrin). Eight replicates were performed for each host plant.

### Statistical analysis

The preferences of aphid for different host plant leaves were analyzed by paired *t*-test. The equality and normality of the variances were determined using the Kolmogorov–Smirnov and Shapiro-Wilk tests for performance assay, EPG results, individual and total amino acid concentration and glucosinolate concentration in the leaves of different host plants. The ln (1 + x) transformation was used to modify any data that did not match the uniformity of variance assumptions. The relationship between nymph produced by adult aphid and adult aphid body weight was analyzed using a linear regression equation. The effect of different host plants on feeding behavior (EPG results), the concentration of individual and total amino acids and glucosinolates in the leaves of different host plants were then analyzed using one-way analyses of variance (ANOVA) followed by Tukey’s honestly significant difference (HSD) post hoc tests at a significance level of *P* < 0.05. All the above analyses were conducted using R 4.2.1 (R Foundation for Statistical Computing, Vienna, Austria) [22]. All figures and graphs were produced in GraphPad Prism v. 9.4.1 for Windows and illustrations were in Adobe Illustrator Package 2021.

## Results

### Preference and performance of *M*. *persicae*

**Fig 1:**
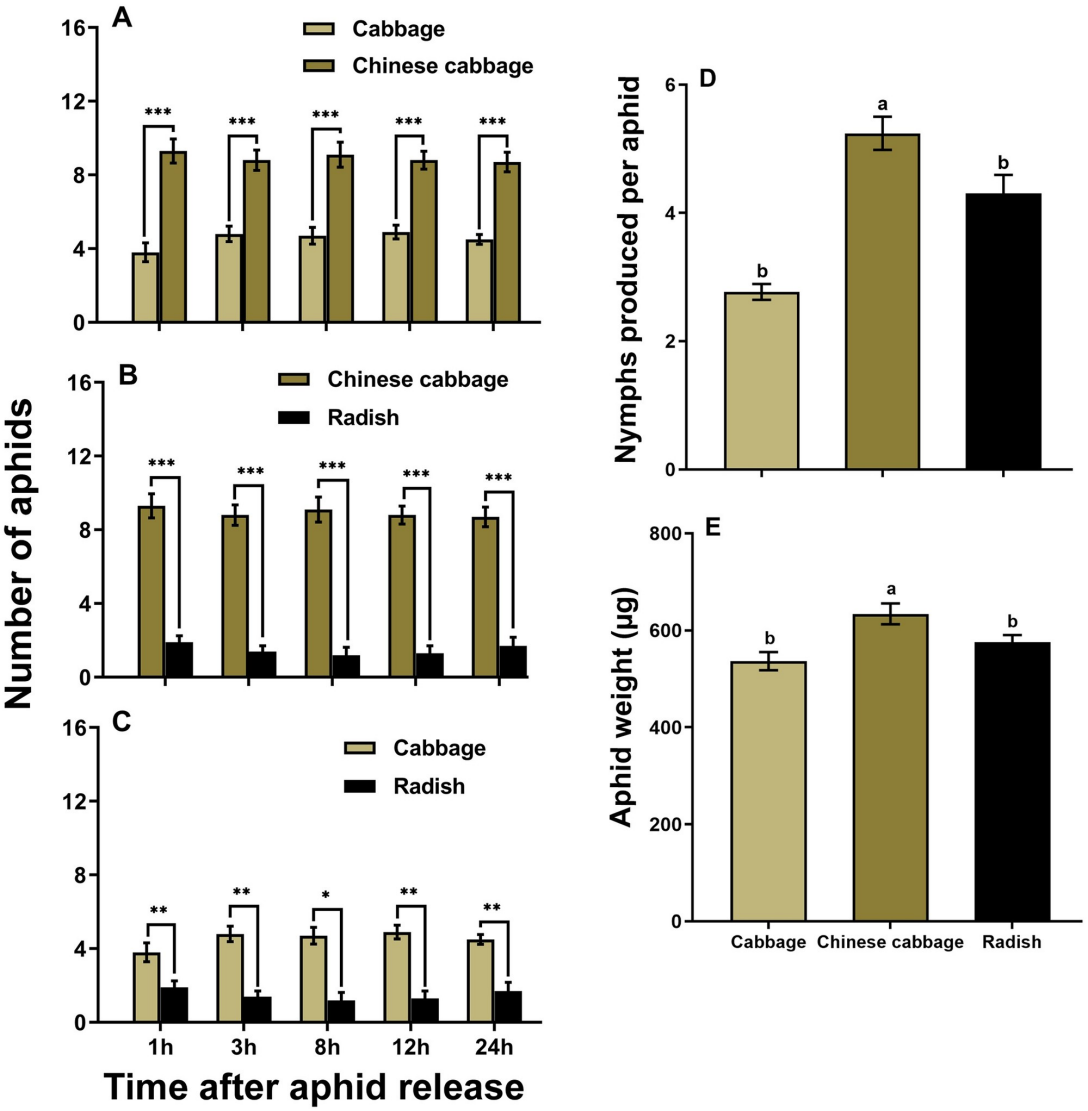
Feeding preference of adult *M*. *persicae* reared on cabbage for different host plant leaves (A-C) (paired *t*-test: * *P* < 0.05, ** *P* < 0.01, *** *P* < 0.001); and growth performance of adult *M*. *persicae* reared on respective plants in terms of nymph production (D); and body weight (E). Values are mean ± SE. Different letters above bars indicate significant differences among plants (one-way ANOVA, Tukey’s HSD test at *P* < 0.05).

Significantly more adult aphids reared on cabbage (Fig 1) as well as Chinese cabbage (see S1 Fig online) and radish (see S2 Fig online) settled on Chinese cabbage leaves compared with cabbage (*t* = 11.59, df = 19, *P* < 0.001; Fig 1A) or radish (*t* = 5.34, df = 19, *P* < 0.001; Fig 1B) plant leaves since 1 h after release. When cabbage and radish plant leaves were tested, significantly more aphids settled on cabbage plant leaves (*t* = 6.24, df = 19, *P* < 0.01; Fig 1C) since 1 h after release. Aphids feeding on Chinese cabbage resulted in significantly more nymph production (*F* = 28.54, df = 2, 27, *P* < 0.001; Fig 1D) and higher adult body weight (*F* = 6.97, df = 2, 27, *P* < 0.05; Fig 1E) than aphids feeding on radish and cabbage. The linear regression results showed that the body weight of adult aphids positively affect the nymph production of adult aphid (R^2^ = 0.2545, *P* < 0.01; see S3 Fig online) when tested among cabbage, Chinese cabbage, and radish plant leaves.

### Aphid feeding behavior

The total probing time of aphids feeding on different host plants was comparative (*P* = 0.957; Table 1). The mean duration of E2 (phloem feeding) (*F* = 5.91, df = 2, 42, *P* < 0.05) and total duration of E2 (*F* = 9.61, df = 2, 42, *P* < 0.05) were significantly shorter when aphids feeding on radish leaves compared with other host plants. No significant difference in the number of E1 (salivation phase) (*P* = 0.194) and E2 (*P* = 0.192) on different host plants was found. Significantly a greater number of probes (*F* = 8.91, df = 2, 42, *P* < 0.001) and short probes (< 3 min) (*F* = 4.32, df = 2, 42, *P* < 0.05) were exhibited by aphids feeding on radish plants (Table 1).

**Table 1:** Probing behavior of *M*. *persicae* on different host plants.

EPG parameters	Cabbage n = 15	Chinese cabbage n = 15	Radish n = 15
Total duration of probing (h)	7.54 a ± 0.23	7.67 a ± 0.09	7.61 a ± 0.45
Total duration of E2 (h)	5.82 a ± 0.34	4.83 a ± 0.62	2.61 b ± 0.57
Mean duration of E2 (h)	3.42 a ± 0.62	1.76 b ± 0.56	1.01 b ± 0.23
Total duration of E1 (min)	5.13 a ± 1.00	4.03 a ± 0.87	3.74 a ± 0.68
Mean duration of E1 (min)	0.94 ab ± 0.16	1.19 a ± 0.15	0.76 b ± 0.04
Number of E2	5.86 a ± 1.05	3.06 a ± 0.95	4.53 a ± 1.18
Number of E1	6.2 a ± 1.06	3.4 a ± 0.94	4.7 a ± 1.19
Number of probes	9.0 b ± 1.70	7.2 b ± 1.74	19.6 a ± 3.02
Number of short probes (< 3 min)	4.46 b ± 1.13	3.4 b ± 1.08	8.8 a ± 1.79
Number of probes to the first E1	4.46 a ± 1.14	6.73 a ± 1.57	7.53 a ± 1.86
Duration of np period before the first E1 (h)	0.07 a ± 0.02	0.20 a ± 0.08	0.18 a ± 0.06
Duration of first probe (min)	119.30 a ± 47.95	49.40 a ± 31.16	25.92 a ± 7.82
Time from first probe to first E1 (h)	0.73 a ± 0.11	1.29 a ± 0.24	1.20 a ± 0.24
Duration of first E (h)	1.77 ab ± 0.76	3.06 a ± 0.75	0.10 b ± 0.02
Total duration of E (h)	4.91 a ± 0.62	5.74 a ± 0.36	2.82 b ± 0.51
Number of single E1	0.20 a ± 0.10	0.33 a ± 0.12	0.26 a ± 0.11
Time from start of EPG to first probe (min)	0.08 a ± 0.05	0.03 a ± 0.00	0.04 a ± 0.01
Time from first probe to first E2 (h)	0.76 a ± 0.11	1.29 a ± 0.23	1.15 a ± 0.24
Time from start of EPG to first E2 (h)	0.77 a ± 0.11	1.28 a ± 0.21	1.24 a ± 0.22
Number of sustained E2 (> 10 min)	2.26 a ± 0.43	1.80 a ± 0.26	1.46 a ± 0.19
Time from start of EPG to first sustained E2 (> 10 min) (h)	2.04 a ± 0.62	1.73 a ± 0.38	2.28 a ± 0.46
Time from first probe to first sustained E2 (> 10 min) (h)	2.03 a ± 0.62	1.63 a ± 0.31	1.99 a ± 0.50
Number of C	12.40 b ± 2.34	7.40 b ± 1.63	24.26 a ± 3.77
Total Duration of C (h)	2.71 a ± 0.57	0.91 b ± 0.19	3.49 a ± 0.50
Mean Duration of C (h)	0.63 a ± 0.31	0.22 a ± 0.03	0.21 a ± 0.04
Number of np	9.00 b ± 1.70	6.86 b ± 1.70	18.53 a ± 2.94
Total duration of np (h)	0.21 b ± 0.05	0.23 b ± 0.08	0.71 a ± 0.12
Mean duration of np (h)	0.01 a ± 0.00	0.02 a ± 0.00	0.03 a ± 0.00
Total duration of no phloem phase (h)	2.84 ab ± 0.57	1.77 b ± 0.28	4.17 a ± 0.62
Duration of the longest E2 (h)	3.66 ab ± 0.6	4.60 a ± 0.45	2.41 b ± 0.52

Data are means ± SE and different letters within each row indicate significant differences (one-way ANOVA, Tukey’s HSD test at *P* < 0.05). E1, salivation; E2, phloem ingestion; np, non-probe; n, number of replicates.

### Amino acid profiles in leaves

**Fig 2:**
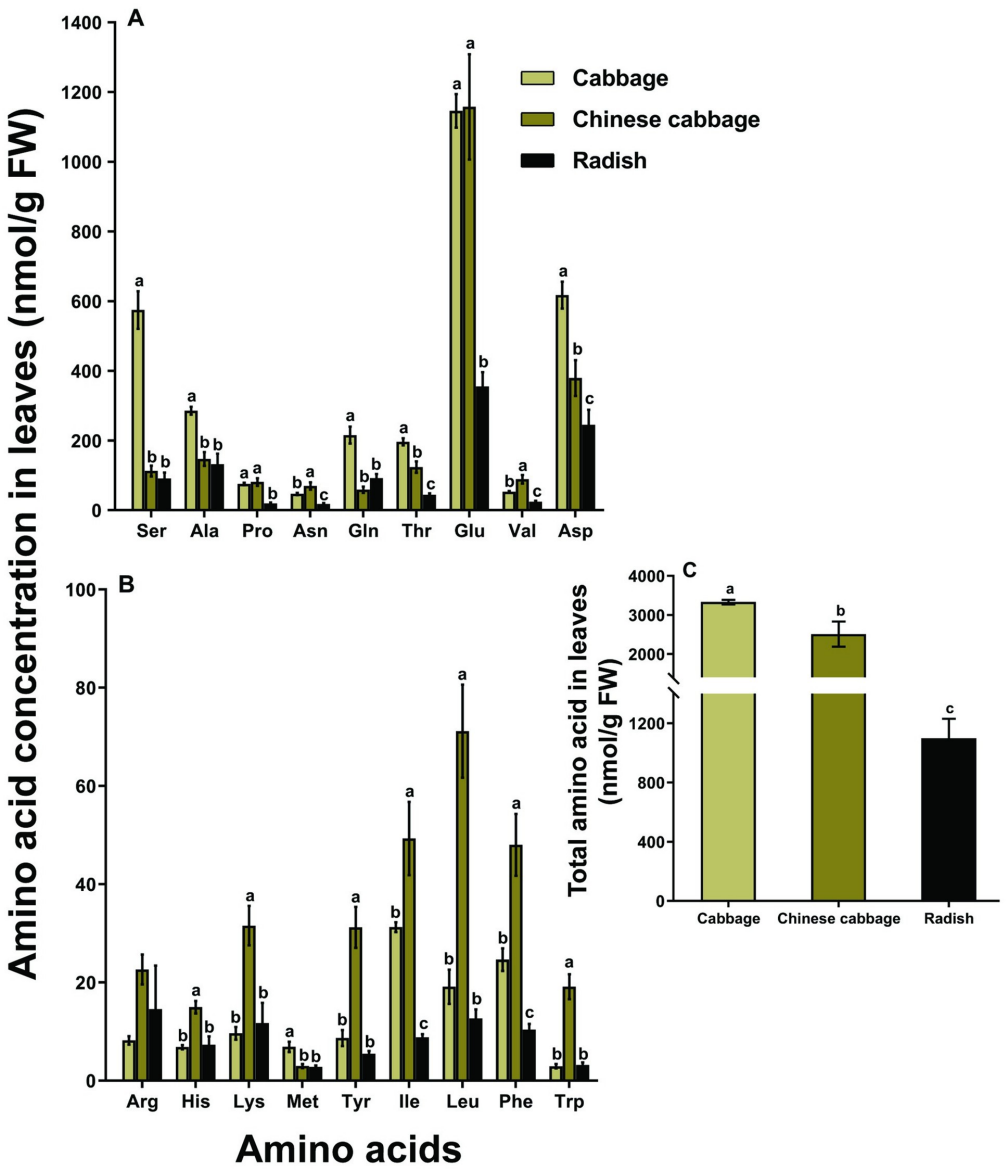
Nutrition quality in the leaves of different host plants. The individual concentration of amino acids (A-B), and the total concentration of amino acids (C). Different letters above bars of each amino acid indicate significant differences among plants (one-way ANOVA, Tukey’s HSD test at *P* < 0.05).

The concentration of the essential amino acid threonine (*F* = 45.34, df = 2, 21, *P* < 0.001), and aspartate (*F* = 17.78, df = 2, 21, *P* < 0.001) in cabbage leaves were significantly higher than Chinese cabbage and radish leaves (Fig 2A). Chinese cabbage leaves contained significantly higher levels of asparagine (*F* = 16.46, df = 2, 21, *P* < 0.001), valine (*F* = 19.67, df = 2, 21, *P* < 0.001), isoleucine (*F* = 21.51, df = 2, 21, *P* < 0.001), and phenylalanine (*F* = 23.27, df = 2, 21, *P* < 0.001) (Fig 2A and 2B). Total amino acid concentration in cabbage or Chinese cabbage leaves was also significantly higher than that in radish leaves (*F* = 31.0, df = 2, 21, *P* < 0.001; Fig 2C).

### Sugar content in leaves

**Fig 3:**
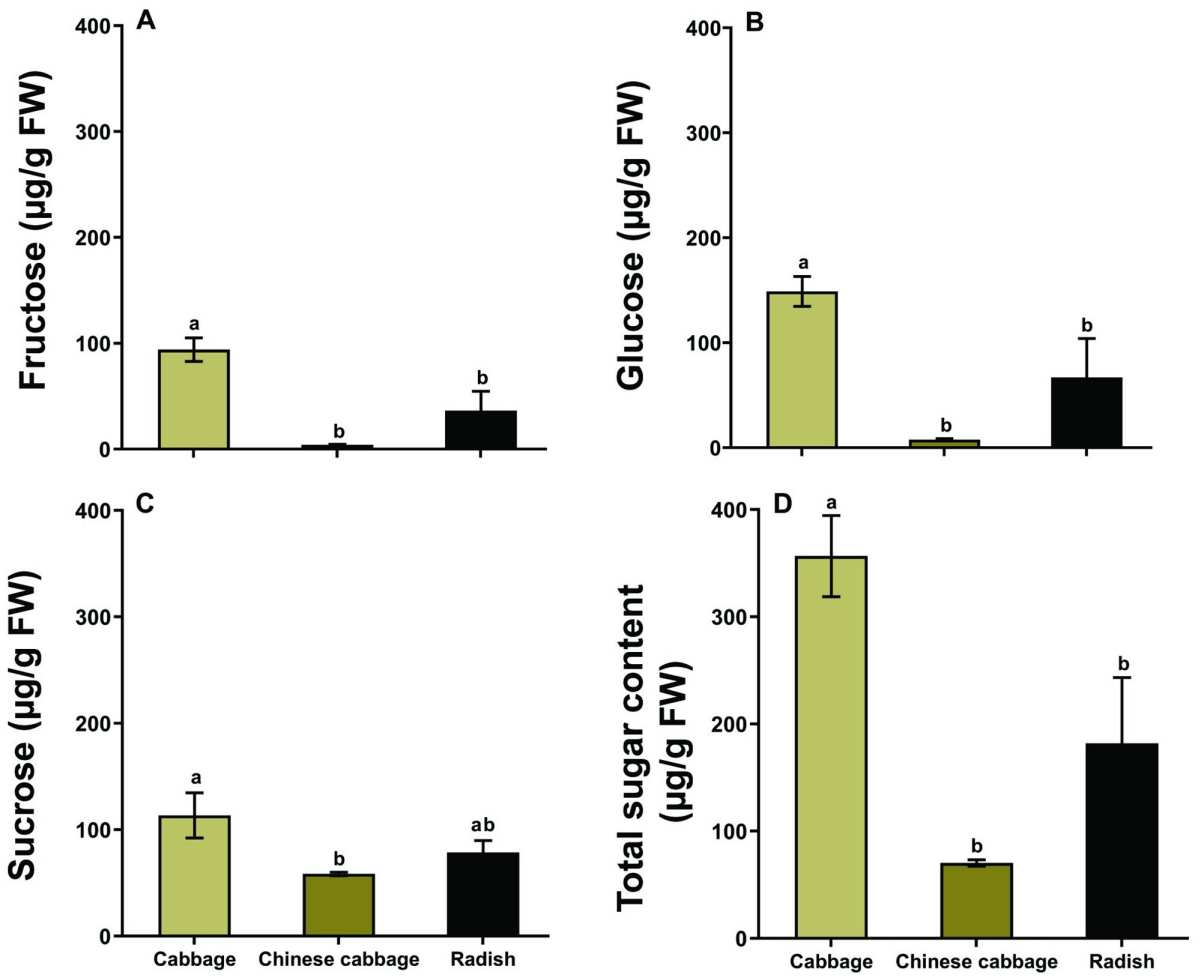
Sugar content in the leaves of different host plants. Fructose (A); glucose (B); sucrose (C); and total sugar content (D). Different letters above bars indicate significant differences (one-way ANOVA, Tukey’s HSD test at *P* < 0.05).

The concentration of fructose (*F* = 13.38, df = 2, 21, *P* < 0.001; Fig 3A), glucose (*F* = 9.51, df = 2, 21, *P* < 0.001; Fig 3B), sucrose (*F* = 4.01, df = 2, 21, *P* < 0.05; Fig 3C), and total sugar (*F* = 12.0, df = 2, 21, *P* < 0.001; Fig 3D) in cabbage leaves were significantly higher than in Chinese cabbage and radish leaves.

### Glucosinolate concentration in leaves

**Fig 4:**
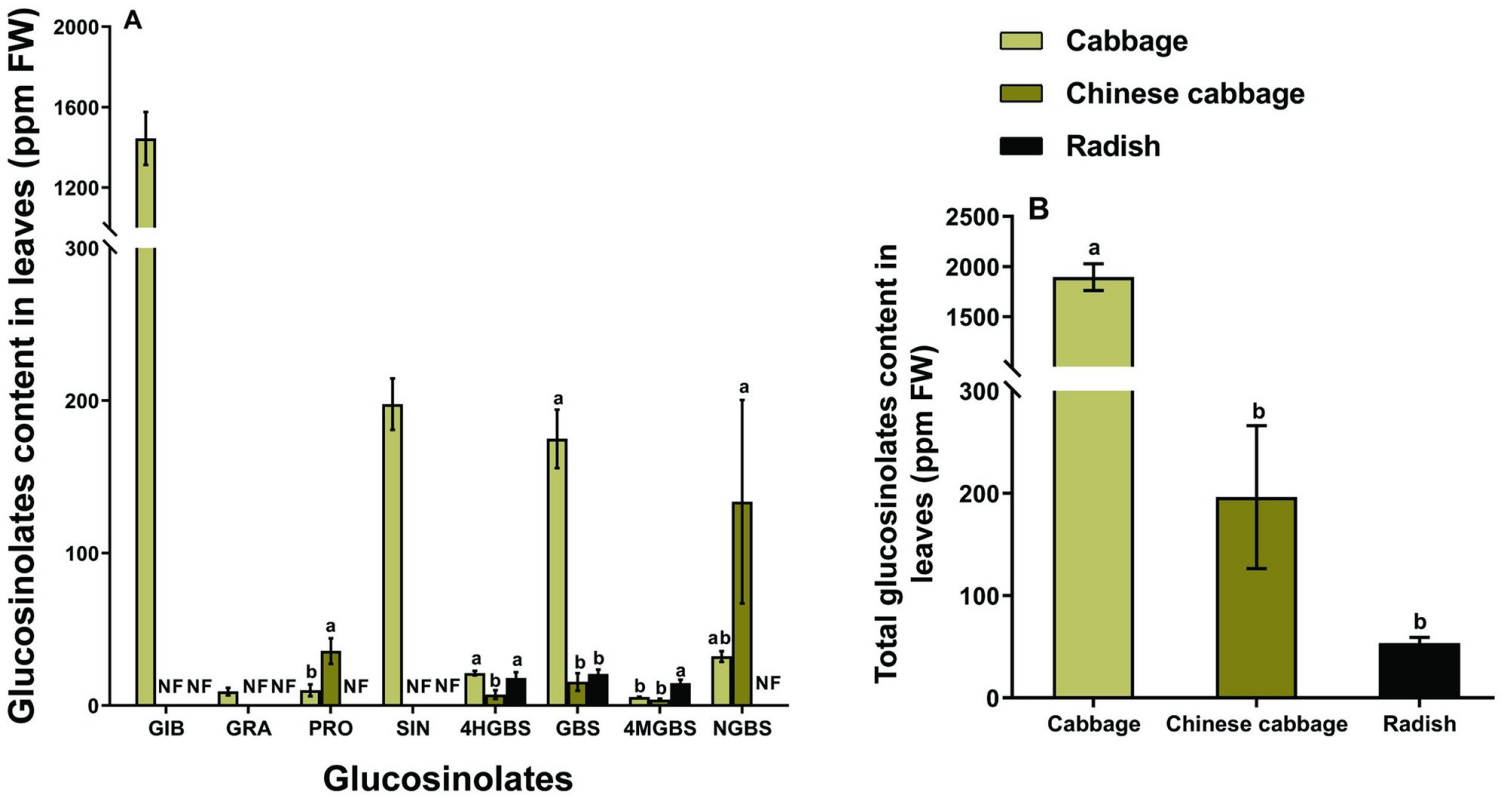
The concentration of glucosinolates in the leaves of different host plants. Individual concentration of glucosinolates (A), and total concentration of glucosinolates (B). Different letters above bars indicate that each glucosinolate is significantly different among plants (one-way ANOVA, Tukey’s HSD test at *P* < 0.05). Glucosinolate side-chain abbreviations: GIB = glucoiberin; GRA = glucoraphanin; PRO = progoitrin; SIN = sinigrin; 4HGBS = 4-hydroxyglucobrassicin; GBS = glucobrassicin; 4MGBS = 4-methoxyglucobrassicin; NGBS = neoglucobrassicin.

The concentrations of glucoiberin (GIB), glucoraphanin (GRA), and sinigrin (SIN) were the highest in cabbage, while these glucosinolates were undetectable in Chinese cabbage and radish leaves. However, the level of progoitrin (PRO) was significantly higher in Chinese cabbage leaves (*F* = 12.05, df = 2, 21, *P* < 0.05; Fig 4A). Indolic glucosinolates 4-hydroxyglucobrassicin (4HGBS), glucobrassicin (GBS), and 4-methoxyglucobrassicin (4MGBS) were found in all three hosts. The concentration of GBS was significantly higher in cabbage leaves (*F* = 59.9, df = 2, 21, *P* < 0.001; Fig 4A), while the concentration of 4MGBS was the highest in radish leaves (*F* = 16.21, df = 2, 21, *P* < 0.001; Fig 4A). Neoglucobrassicin (NGBS) with the highest concentration was found in the Chinese cabbage leaves (Fig 4A). The total content of glucosinolates in cabbage leaves was about ten times and 40 times higher than Chinese cabbage and radish leaves, respectively (*F* = 140.0, df = 2, 21, *P* < 0.001; Fig 4B).

## Discussion

Our results showed that *M*. *persicae* preferred and performed better on Chinese cabbage, which may be partly due to the higher level of nutrient (amino acids) and lower level of glucosinolates in the Chinese cabbage leaves. In contrast, the higher concentration of glucosinolates in cabbage and lower level of amino acids in radish may account for the poorer preference and growth of *M*. *persicae* on these two plants, respectively. These results suggest that both amino acids and glucosinolates in Brassicaceae plants influence the preference and performance of *M*. *persicae* which is in accordance with previous publications [10,23,24]. In most cases, aphids make their decision to accept the host plant before their stylet reaches phloem sieve element, relying on nutrients or toxins present in the peripheral (non-vascular) plant cells [25]. In the feeding preference assay, significantly more aphids settled on Chinese cabbage leaves since 1 h after release; however, EPG data showed that *M*. *persicae* took more than 1 h to reach the phloem (E1), indicating that factors that influence aphid feeding host preference may be located at epidermal or mesophyll cells [25]. Therefore, in this study, the variation of amino acids and glucosinolates in plant leaves may, at least partly, account for the difference of feeding preference of *M*. *persicae*.

Compared with cabbage, Chinese cabbage leaves contained relatively lower levels of feeding stimulants (sucrose) and comparable amino acids; thus, more aphids selected Chinese cabbage possibly because of the lower glucosinolate contents in Chinese cabbage leaves. Indole glucosinolates has been shown strong antixenosis effects against *M*. *persicae*, and we found that the indole glucosinolates 4HGBS and GBS in Chinese cabbage leaves were lower than those in cabbage leaves (Fig 4A) [13,15]. Stimulatory and defensive metabolites as well as the characteristics of leaf surface and composition of the plant cell wall may influence the feeding preference of *M*. *persicae* [7,25], however, *M*. *persicae* does not usually accept or reject brassica host plants due to phagostimulants at the leaf surface or from cell penetrations along the stylet pathway, unless the glucosinolate concentration is very high [26]. The shorter mean phloem-feeding duration (E2) of *M*. *persicae* feeding on Chinese cabbage or radish leaves suggested that aphids repeatedly pull out their stylets from the phloem of these leaves. The total probing time and time from the first probe to the first phloem (E1) of aphids fed on three plants were not significantly different, suggesting that the leaf surface or peripheral (nonvascular) plant cells of these plants may not affect aphid feeding preference.

Amino acids and glucosinolates are the key nutrition and defensive metabolites respectively, in the Brassicaceae plants that determine aphid performance [3,27,28], however, there are few exceptions [29,30]. In this study, there was no significant difference in amino acid content between cabbage and Chinese cabbage. In addition, although the mean phloem feeding time was significantly different between Chinese cabbage and cabbage, the total time was not, suggesting that aphids obtain a similar amount of nutrition from these two plants. Thus, the significantly higher level of glucosinolates in cabbage leaves may account for the lower body weight and fecundity of *M*. *persicae* on this plant. This hypothesis is in accordance with a previous study where results suggested that the glucosinolates profiles have a significant impact on the development and performance of both *B*. *brassicae* and *M*. *persicae* on wild and cultivated brassica species [31]. However, in some previous studies, *M*. *persicae* grew better on young cabbage leaves or pre-infested Chinese cabbage leaves that contain a higher level of glucosinolates, which could be explained by that aphids also ingested considerably more nutrition (mainly the amino acids) from these leaves [8,10,32]. Although radish contained a lower level of glucosinolates, it also had fewer nutrients, which may be the reason why aphids had poorer growth and preference on this plant. In addition, aphids had a shorter phloem sap time on radish and thus ingested fewer nutrients when feeding on radish. These results suggest that the effect of glucosinolates in plant resistance against aphids is highly variable, which could be affected by aphid’s detoxification ability or nutrient compensation [6,10]. Nevertheless, we cannot exclude the possibility that some other unknown factors may also be involved in this plant-aphid interaction.

This study reveals that *M*. *persicae* prefers and performs better on Chinese cabbage, which may partly be due to both the nutrients and defensive metabolites in the host plants. Our results suggest that Chinese cabbage is more susceptible to *M*. *persicae* and may need more field monitoring and control measures to manage this pest and will also provide new knowledge for breeding aphid resistant Brassicaceae crops.

## Supporting information

S1 Fig Feeding preference of adult *M*. *persicae* for different host plant leaves (A-C) (paired *t*-test: * *P* < 0.05, ** *P* < 0.01, *** *P* < 0.001) reared on Chinese cabbage plant. Values are mean ± SE.
(DOCX)

S2 Fig Feeding preference of adult *M*. *persicae* for different host plant leaves (A-C) (paired *t*-test: * *P* < 0.05, ** *P* < 0.01, *** *P* < 0.001) reared on radish plant. Values are mean ± SE.
(DOCX)

S3 Fig The relationship between adult aphid body weight and nymph produced per aphid on different host plants. R2 values show a negative relationship among adult aphid body weight and nymph production in the slope from zero (black dot means aphids on cabbage, red square means aphids on Chinese cabbage, and blue triangle means aphids on radish).
(DOCX)
