# Supplementary material for: Preference and performance of the green peach aphid, Myzus persicae on three Brassicaceae vegetable plants and its association with amino acids and glucosinolates
Source: PLoS One. 2022 Dec 1;17(12):e0269736. doi: 10.1371/journal.pone.0269736 (PMC9714699; doi:10.1371/journal.pone.0269736)
Supplement: S2 Fig — Values are mean ± SE. (DOCX) [file pone.0269736.s002.docx]

**Preference and performance of the green peach aphid, *Myzus persicae* on three Brassicaceae vegetable plants and its association with amino acids and glucosinolates**

Muhammad Afaq Ahmed^1,2^, Ning Ban^1^, Sarfaraz Hussain^3^, Raufa Batool^2^, Yong-Jun Zhang^2^, Tong-Xian Liu^1*^,He-He Cao^1*^

**1** Key Laboratory of Insect Ecology and Molecular Biology, College of Plant Health and Medicine, Qingdao Agricultural University, Qingdao, Shandong, China

**2** State Key Laboratory for Biology of Plant Diseases and Insect Pests, Institute of Plant Protection, Chinese Academy of Agricultural Sciences, Beijing, China

**3** Key Laboratory of Agro-products Quality and Safety Control, Institute of Food Science and Technology, Chinese Academy of Agricultural Sciences, Beijing, China


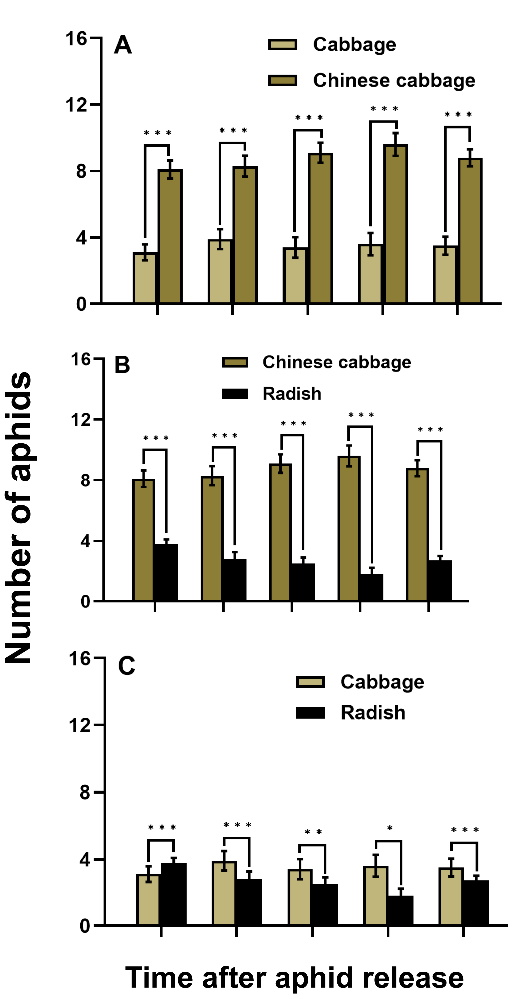


**1 h**

**3 h**

**8 h**

**12 h**

**24 h**

**Time after aphid release**

**Number of aphids**

**S2 Fig. Feeding preference of adult *M. persicae* reared on radish plant for different host plant leaves (A-C) (paired *t*-test: * *P* < 0.05, ** *P* < 0.01, *** *P* < 0.001). Values are mean ± SE.**
