# Supplementary material for: Preference and performance of the green peach aphid, Myzus persicae on three Brassicaceae vegetable plants and its association with amino acids and glucosinolates
Source: PLoS One. 2022 Dec 1;17(12):e0269736. doi: 10.1371/journal.pone.0269736 (PMC9714699; doi:10.1371/journal.pone.0269736)
Supplement: S3 Fig — R2 values show a negative relationship among adult aphid body weight and nymph production in the slope from zero (black dot means aphids on cabbage, red square means aphids on Chinese cabbage, and blue triangle means aphids on radish). (DOCX) [file pone.0269736.s003.docx]

**Preference and performance of the green peach aphid, *Myzus persicae* on three Brassicaceae vegetable plants and its association with amino acids and glucosinolates**

Muhammad Afaq Ahmed^1,2^, Ning Ban^1^, Sarfaraz Hussain^3^, Raufa Batool^2^, Yong-Jun Zhang^2^, Tong-Xian Liu^1*^,He-He Cao^1*^

**1** Key Laboratory of Insect Ecology and Molecular Biology, College of Plant Health and Medicine, Qingdao Agricultural University, Qingdao, Shandong, China

**2** State Key Laboratory for Biology of Plant Diseases and Insect Pests, Institute of Plant Protection, Chinese Academy of Agricultural Sciences, Beijing, China

**3** Key Laboratory of Agro-products Quality and Safety Control, Institute of Food Science and Technology, Chinese Academy of Agricultural Sciences, Beijing, China


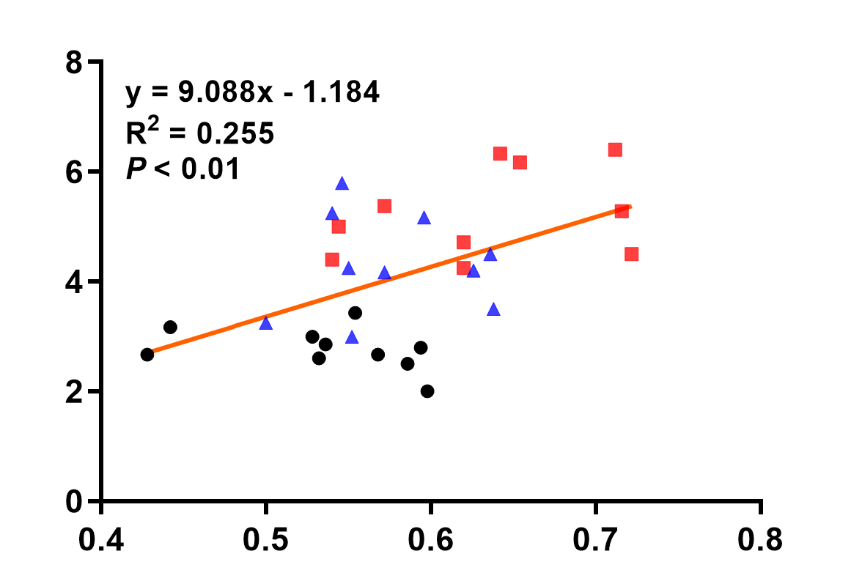


**Adult aphid body weight (mg)**

**Nymph produced per aphid**

**S3 Fig. The relationship between adult aphid body weight and nymph produced per aphid on different host plants. R^2^ values show a negative relationship among adult aphid body weight and nymph production in the slope from zero (black dot means aphids on cabbage, red square means aphids on Chinese cabbage, and blue triangle means aphids on radish).**
